# Cerebellar lobules and dentate nuclei mirror cortical force‐related‐BOLD responses: Beyond all (linear) expectations

**DOI:** 10.1002/hbm.23541

**Published:** 2017-02-27

**Authors:** Adnan A.S Alahmadi, Matteo Pardini, Rebecca S. Samson, Karl J. Friston, Ahmed T. Toosy, Egidio D'Angelo, Claudia A.M. Gandini Wheeler‐Kingshott

**Affiliations:** ^1^ Department of Diagnostic Radiology Faculty of Applied Medical Science King Abdulaziz University (KAU) Jeddah Saudi Arabia; ^2^ NMR Research Unit, Department of Neuroinflammation Queen Square MS Centre UCL Institute of Neurology London United Kingdom; ^3^ Department of Neurosciences Rehabilitation, Ophthalmology, Genetics and Maternal and Child Health, University of Genoa Genoa Italy; ^4^ Wellcome Trust Centre for Human Neuroimaging, UCL, Institute of Neurology London United Kingdom; ^5^ Brain Connectivity Centre, C. Mondino National Neurological Institute Pavia Italy; ^6^ Department of Brain and Behavioural Sciences University of Pavia Italy; ^7^ Brain MRI 3T Mondino Research Center C. Mondino National Neurological Institute Pavia Italy

**Keywords:** FMRI, force, non‐linearity, cerebellum, power grip, SUIT, dentate‐nuclei

## Abstract

The relationship between the BOLD response and an applied force was quantified in the cerebellum using a power grip task. To investigate whether the cerebellum responds in an on/off way to motor demands or contributes to motor responses in a parametric fashion, similarly to the cortex, five grip force levels were investigated under visual feedback. Functional MRI data were acquired in 13 healthy volunteers and their responses were analyzed using a cerebellum‐optimized pipeline. This allowed us to evaluate, within the cerebellum, voxelwise linear and non‐linear associations between cerebellar activations and forces. We showed extensive non‐linear activations (with a parametric design), covering the anterior and posterior lobes of the cerebellum with a BOLD‐force relationship that is region‐dependent. Linear responses were mainly located in the anterior lobe, similarly to the cortex, where linear responses are localized in M1. Complex responses were localized in the posterior lobe, reflecting its key role in attention and executive processing, required during visually guided movement. Given the highly organized responses in the cerebellar cortex, a key question is whether deep cerebellar nuclei show similar parametric effects. We found positive correlations with force in the ipsilateral dentate nucleus and negative correlations on the contralateral side, suggesting a somatotopic organization of the dentate nucleus in line with cerebellar and cortical areas. Our results confirm that there is cerebellar organization involving all grey matter structures that reflect functional segregation in the cortex, where cerebellar lobules and dentate nuclei contribute to complex motor tasks with different BOLD response profiles in relation to the forces. *Hum Brain Mapp 38:2566–2579, 2017*. © **2017 Wiley Periodicals, Inc.**

## INTRODUCTION

The ability to grip using the hands is one of the key functions allowing primates to use tools to act on the proximal environment. Object gripping is a complex motor act, because it requires the integration of visuo‐spatial and proprioceptive information, correct application, timing of different grip forces (GF) and coordination with other concurrent motor activities [Ulloa et al., 2003]. The cerebellum has been shown to be involved in all of these facets of motor control, using blood‐oxygen‐level‐dependent (BOLD) signals measured with functional magnetic resonance imaging (fMRI) [Stoodley, 2012; Stoodley and Schmahmann, 2009]. The signals have also been seen in the initiation of grip movements, in association with the other key areas of the motor system. Generally, there are two types of gripping [King et al., 2014; Napier, 1956]: “Power” where all of the fingers of the hand participated in generating the movement (i.e., gripping an object) and “precision” where the thumb and at least one other finger perform the gripping. These two types of gripping can be performed either statically or dynamically (i.e., patterns of gripping) as explained by Landsmeer [1962] and King et al. [2014] where they considered dynamic movement is repeated in cycles of up to 3.5 sec and static movement where the gripping is held constantly for a period of time longer than 3.5 sec.

Many neuroimaging studies test for linear relationships between signals that reflect activity at the neuronal level and experimental factors (e.g., forces) [Ashe, 1997; Dettmers et al., 1996; Ehrsson et al., 2001; Halder et al., 2007; Kuhtz‐Buschbeck et al., 2001, 2008; van Duinen et al., 2007], while the nature and meaning of non‐linear relationships—of the sort characterized by *psychometric* functions in psychophysics—have received less attention. Several reasons may explain this, including difficulties in interpretation and also, importantly, detecting them. The rationale for our study of non‐linear *neurometric* functions is based on the non‐linearity inherent in neuronal dynamics and synaptic transmission, which define the relationship between neural activity and behavior; often found to be non‐linear—as shown by multiple neurophysiological studies [Ashe, 1997; Conrad et al., 1977; Cheney and Fetz, 1980; Evarts, 1967; Evarts et al., 1983; Georgopoulos et al., 1992; Hepp‐Reymond et al., 1994; Maier et al., 1993; Taira et al., 1996]. If we consider the experimental factors of an fMRI experiment, the relationship with BOLD responses is further complicated by non‐linearities in neurovascular coupling [Friston et al., 2000], which has been specifically analyzed in the cerebellum [Mapelli et al., 2017]. These considerations demonstrate the need to characterize non‐linear responses in the healthy human brain. In support of our claims, non‐linear associations between input task and fMRI signal were also detected when using the non‐dominant hand to perform the same task in healthy controls as well as in multiple sclerosis patients (Alahmadi et al. 2016a; Alahmadi et al. 2016b; Alahmadi et al. 2016c) motivating the need for investigating these patterns further. The outcome of our analysis may help to inform future studies aimed at understating the physiological basis of these neurometric functions.

To date, however, the relative contributions of the different cerebellar lobules to gripping tasks (using fMRI) and the precise relationship between varying GFs and cerebellar activity have not been studied in detail apart from one careful study by Spraker et al. [2012] where the authors used a static precision grip task. This contrasts with previous analyses at the whole brain level [Alahmadi et al., 2015, 2016d; Ehrsson et al., 2000; Halder et al., 2007; Keisker et al., 2009, 2010; King et al., 2014; Kuhtz‐Buschbeck et al., 2001, 2008; Neely et al., 2013; Noble et al., 2013; Spraker et al., 2012; Vaillancourt et al., 2003; Ward and Frackowiak, 2003]. Some of these studies report a linear association between GF strength and areas of the anterior cerebellum [Halder et al., 2007; Kuhtz‐Buschbeck et al., 2008] but not in the posterior cerebellum, despite the role of the posterior cerebellum in visual processing (when using visual cues) and motor planning [King et al., 2014; Stoodley and Schmahmann, 2010; Stoodley et al., 2012]. Conversely, a recent dynamic power grip study found that the signal in the anterior cerebellum was linearly correlated with forces, whereas the posterior cerebellum evidenced higher signals at low and high forces but low signals at intermediate force levels [Keisker et al., 2009]. In contrast with all of these findings, a static precision grip fMRI study found that the signals in the whole (i.e., anterior and posterior) cerebellum tended to covary non‐linearly with force [Spraker et al., 2012].

Two issues are clear from reviewing the literature: there seems to be some difficulty in detecting fMRI signals in the cerebellum and, when detected, there is little agreement on the relationship between GF and BOLD signal in the cerebellum. Different explanations may underlie the heterogeneity of published results on this topic, including differences in the types of grip task (power vs. precision), in grip patterns (static vs. dynamic) and importantly, the number and ranges of force levels used in the experimental setting [Alahmadi et al., 2016d; Keisker et al., 2010; King et al., 2014; Kuhtz‐Buschbeck et al., 2008].

In a recent visuomotor study [Alahmadi et al., 2016d], we investigated the relationship between whole brain activations and GF using five force levels and a dynamic power grip task in right‐handed healthy subjects. We showed that in cortical and sub‐cortical areas, the BOLD signal tends to vary linearly or non‐linearly in different motor and non‐motor regions including the sensorimotor cortex (M1/S1), the supplementary motor area (SMA), the superior and inferior parietal lobules (SPL and IPL), insula, and several visual and associative areas. The analysis performed in that study, however, was not specifically aimed at investigating the cerebellum: as such, we only observed the main effects of gripping in the anterior and part of the posterior cerebellum and only a non‐linear (4th order polynomial) relationship between GFs and contralateral cerebellum hemisphere activations (located in lobule VI).

The fMRI analysis pipeline, optimized for whole brain analysis, however, is known to lead to suboptimal results in the cerebellum [Diedrichsen, 2006]. Given the known visuomotor, sensory, associative and cognitive functions of the cerebellum [Stoodley, 2012; Stoodley and Schmahmann, 2009, 2010], a cerebellum‐specific fMRI analysis that uses the spatially unbiased infratentorial template (SUIT) software for the cerebellum was applied to the same data set as in Alahmadi et al. [2016d], to provide a detailed characterization of parametric cerebellar responses to GF. SUIT has been shown to improve localization, statistical inferences, and normalization in the cerebellum [Diedrichsen, 2006]. Compared to the Montreal Neurological Institute whole brain template, Diedrichsen showed that the alignment of individual cerebellum fissures was improved by 60%, the statistical inferences were increased by up to 15% and the activated volumes and *t*‐values increased similarly. Here, the SUIT and dentate nuclei (DN) templates [Diedrichsen, 2006; Diedrichsen et al., 2011] were applied for the first time to study a dynamic power grip task to exploit and accurate anatomical co‐localization of these regions.

Our study hypothesis was that the signal responses in the cerebellum would show extensive effects of GF, similar to those reported for the neo‐cortex, displaying: linear positive response to GF in motor regions, higher order non‐linear (parametric) forms in regions known to be involved in sensory, associative and cognitive processes, and negative response in visual areas as well as in motor areas. Furthermore, as the deep cerebellar nuclei are also key to motor and associative processing [Dimitrova et al., 2006; Gao et al., 1996; Habas, 2010; Küper et al., 2011, 2012, 2014], and their activity is modulated by the complexity of visuomotor processes [Alahmadi et al., 2015], we expected that the DN may also respond in a non‐linear manner, according to patterns typical of associative areas of the cerebellar cortex.

## METHODS

The subjects and acquisition protocol are the same as those described in [Alahmadi et al., 2016d], where whole brain analyses were reported. The analysis protocol has been modified here to focus on the cerebellum and DN according to [Diedrichsen, 2006; Diedrichsen et al., 2011].

### Subjects

Thirteen right‐handed healthy volunteers with no history of neurological disease (5 female, 8 male; mean age 31 (± 4.64) years) participated in this study. The handedness of subjects was tested using the Edinburgh handedness scaling questionnaire [Oldfield, 1971]. All participants gave informed consent and the local research and ethics committee approved the study.

### MRI Protocol

A 3.0 T MRI scanner (Philips Achieva, Philips Healthcare, Best, The Netherlands) with a 32‐channel head coil was used. The imaging protocol comprised: A BOLD sensitive T2*‐weighted echo planar imaging (EPI) sequence: echo time (TE)/repetition time (TR) = 35/2,500 ms, voxel size = 3 × 3 × 2.7 mm^3^, inter‐slice gap of 0.3 mm, SENSE = 2, number of slices = 46 acquired with descending order, FOV = 192 × 192 mm^2^, number of volumes = 200, flip angle = 90°, and a three dimensional (3D) anatomical T1‐weighted reference scan (3DT1): 3D inversion‐recovery prepared gradient‐echo (fast field echo) sequence with inversion time (TI) = 824 ms, TE/TR = 3.1/6.9 ms, flip angle = 8°, and voxel size = 1 mm isotropic.

### FMRI Paradigm

Subjects performed a dynamic power grip task with their right (dominant) hand, using an MR‐compatible squeeze ball [see Alahmadi et al., 2016d]. Compression of the ball results in an air pressure measurement proportional to the force exerted. An event‐related paradigm was optimized using OptSeq (http://www.surfer.nmr.mgh.harvard.edu/optseq) and comprised 75 active trials divided equally into 5 GF targets (20, 30, 40, 50, and 60% of each subject's maximum voluntary contraction [MVC]) interleaved with 75 rest trials. Each active trial lasted 3 sec and trials were specified in a randomized order. A visual cue was used to instruct the subject, defining the target force and providing interactive (live) feedback on the subject's performance for each trial [figure 1 in Alahmadi et al., 2016d]. The visual cue was a black bar indicating the GF level that subjects needed to reach, while the subject's performance was indicated by a green column—that reached the black line (or a red one for an overshoot).

**Figure 1:**
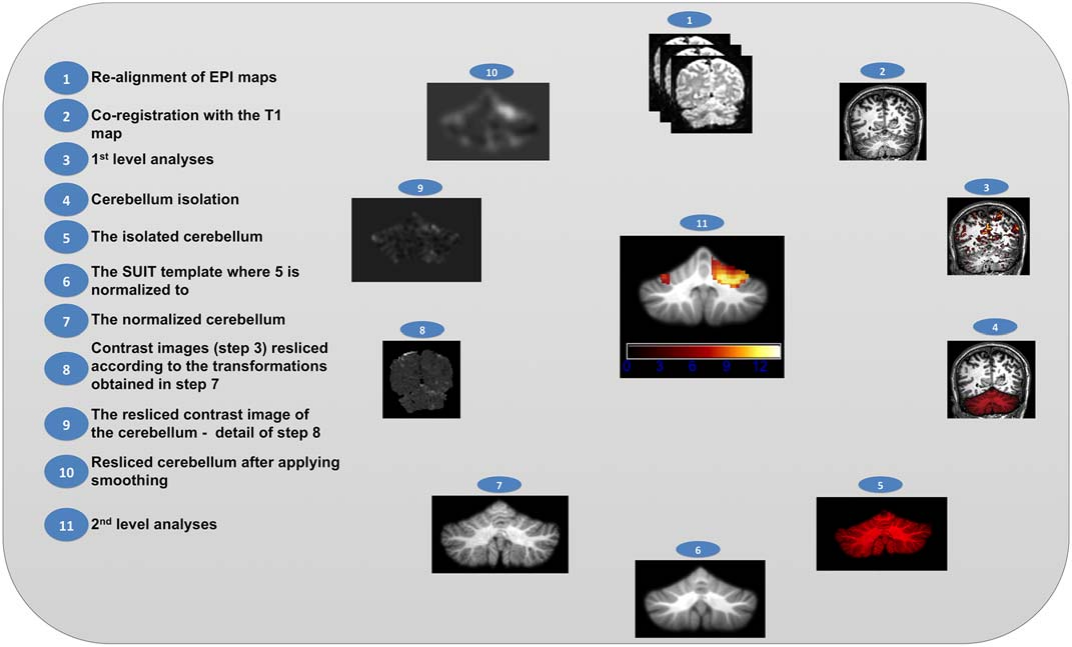
A flowchart summarizing the cerebellar analysis performed using SUIT as described in the steps 1–11 above. [Color figure can be viewed at http://wileyonlinelibrary.com]

### Image Pre‐Processing and Statistical Analyses

Figure 1 shows the processing pipeline for the analysis of cerebellar responses. The pre‐processing steps for each subject followed an fMRI analysis procedure specifically designed for analysis of the cerebellum, guided by the SUIT, part of the statistical parametric mapping software package (SPM12) [Diedrichsen, 2006]. The SUIT template was used to optimize normalization procedures specific to the cerebellum. Pre‐processing for the functional maps included slice timing, realignment, and co‐registration of the EPI fMRI to the 3D T1‐weighted volumes. Then, the within‐subject (first level) analysis was performed for each subject. Given the short GF trial duration (3 sec), which is less than the time constant of the haemodynamic response function (HRF), all GF responses were specified as delta functions [Friston et al., 1998]. Each first level design matrix model included five regressors of interest that comprised polynomial functions of GF of increasing orders (up to the 4th order) [Buchel et al., 1996, 1998]. These functions can capture most (smooth) non‐linear responses [Alahmadi et al., 2016d]. The neurometric functions described by the polynomial expansions describe changes in amplitude of the BOLD response as a function of GF. The resulting parameter estimates or polynomial coefficients represent the change of the BOLD signal per unit change of the polynomial function. In our case, this would be BOLD/MVC %. The 0th‐order term represents the main effect of hand gripping compared to the rest condition, irrespective of the applied GF. The 1st‐order term models linear BOLD changes with force level; higher order non‐linear effects are modelled by subsequent regressors, and accommodate parametric non‐linear shapes (e.g., the U‐shaped captured by the positive 2nd order or more complicated neurometric functions that can be approximated by the 3rd and 4th polynomial orders). It should be noted that non‐linear effects mean that the BOLD signal has a non‐linear dependency on GF. The non‐linear terms include any order above (linear) 1st order effects. Each polynomial regressor was convolved with the canonical HRF to create a standard general linear model (GLM) [Friston et al., 1995, 1998]. Head movement parameters were included in the GLM as regressors of no interest [Friston et al., 1996]. At this level and for the whole brain data, *t*‐statistics were used to test for the effects of each polynomial coefficient to generate contrast images and statistical maps for each polynomial order and for each subject that can then be used for the second level analysis. Then, the following SUIT steps were performed: (1) Extraction of each subject's cerebellum and brainstem from their corresponding whole brain 3DT1 anatomical image; (2) Normalization of the anatomical images to the SUIT template using a non‐linear deformation; (3) Re‐slicing of the functional contrast images produced from the first level analysis using the deformation produced from step 2 and masking out any activation outside the region of interest (i.e., the cerebellum). The normalized cerebellum functional contrast images (of each polynomial order) from each subject were then smoothed with an 8mm isotropic full‐width half maximum (FWHM) Gaussian kernel and submitted to a (between‐subjects) standard second level random effects analysis, testing for increasingly higher order non‐linear effects within the cerebellum with one sample *t*‐test. In other words, the images of polynomial coefficients were then used as summary statistics for between subject (second level) random effects analysis (i.e., tests of the null hypothesis using *t*‐tests to create SPMs in the usual way). Significance was set at a corrected (FWE) cluster level using a threshold of *P* < 0.001; minimum extent ten voxels. This threshold corresponds to a *t*‐value of 3.92. The anatomical identification of the activations was defined using a high resolution probabilistic atlas defined by the SUIT template [Diedrichsen et al., 2009]. The activation maps were projected on to the flat map of the cerebellum provided with the SUIT template [Diedrichsen and Zotow, 2015].

In addition, signals from the DN, which appears hypointense on T2*‐weighted images, were analyzed differently as described in [Diedrichsen et al., 2011; Küper et al., 2011]. To prevent functional activations being smoothed into the DN, the functional maps were masked with the DN anatomical regions and a smoothing kernel of 4mm isotropic FWHM was applied [Diedrichsen et al., 2011; Küper et al., 2014]. We also sub‐divided the DN according to Küper et al., 2011 into four regions: dorso‐rostral (DRDN), dorso‐caudal (DCDN), ventro‐rostral (VRDN), and ventro‐caudal (VCDN). It should be noted that alternative terminology could be: Anterior = Rostral; Posterior = Caudal; Superior = Dorsal; Inferior = Ventral. Full details of how to analyze the DN using SUIT are provided in the above articles; for explanations of the methods, see [Diedrichsen et al., 2011] and for specific applications, see [Küper et al., 2011, 2012, 2014]. Significance was set at a corrected (FWE) cluster level using a threshold of *P* < 0.001.

Moreover, to highlight the predominant polynomial order effect from the force‐BOLD fitting at the single voxel level, we categorized the force related effects according to the greatest effect size (among the four polynomial orders) [Alahmadi et al., 2016d].

In addition, to enhance the quality check of the data, we inspected the effects of head motion both visually and quantitatively. We used the Artifact Detection Tools (ART) (http://www.nitrc.org/projects/artifact_detect) to aid this analysis and help detect any obvious outliers in the functional volumes for each participant.

## RESULTS

All subjects were able to perform the task adequately as previously reported [Alahmadi et al., 2016d] (Table 1).

**Table 1:** Task performance showing average (±SD) MVC (%) and duration for each grip force applied during the task

	20%	30%	40%	50%	60%
MVC %	21.61 ± 2.13	30.04 ± 2.04	39.65 ± 1.67	46.99 ± 1.45	57.56 ± 2.44
Duration (s)	2.65 ± 0.59	2.85 ± 0.11	2.78 ± 0.26	2.90 ± 0.09	2.99 ± 0.08

**Figure 2:**
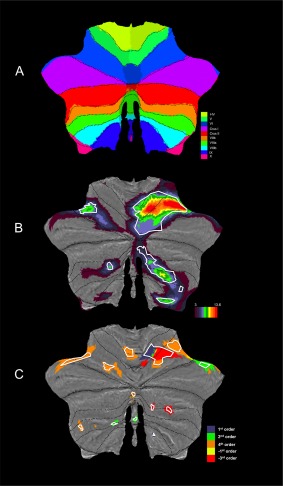
Anatomical identification of cerebellar regions (**A**); effects of the 0th order form (**B**); effects of forcerelated forms (**C**). All effects are projected onto the SUIT flattened map. In the map, right is right (ipsilateral) and the threshold for (normalized) effect sizes were set at *t*‐value > 3, for display purposes only (significant results are discussed in the text at a corrected level of *t*‐value > 3.92. These are shown as white contours in the figure). Note, that the map was thresholded across all force related effects by the largest effect size. [Color figure can be viewed at http://wileyonlinelibrary.com]

For the main effect of movement (i.e., the 0th‐order effect), activations were found in the ipsilateral lobules (I‐VIII); as well as the contralateral lobule (VI) and part of the anterior and posterior vermis (Fig. 2). Table 2 reports all the activated regions for the 0th order effect.

**Table 2:** Activations (maximum significant voxels (foci) for each cluster) for all the fitted polynomial orders along with their anatomical regions and the % probability of involving a certain area

*T*	*x*	*y*	*z*	Anatomical region	%
**0th order**					
13.55	16	−56	−23	Right Cerebellum	VI (Hem) (92); V (5)
13.19	28	−52	−27	Right Cerebellum	VI (95)
10.48	12	−60	−13	Right Cerebellum	VI (Hem) (87); V (13)
7.07	20	−74	−19	Right Cerebellum	VI (Hem) (57); VIIa Crus I (Hem) (4)
6.57	2	−74	−15	Cerebellar Vermis	VI (Vermis) (100)
10.23	22	−62	−47	Right Cerebellum	VIIIa (Hem) (17); VIIIb (Hem) (2)
7.16	12	−74	−45	Right Cerebellum	VIIb (Hem) (50); VIIIa (Hem) (31); (VIIIa (Vermis) (4);
					VIIa Crus II (Hem) (4)
5.81	6	−70	−35	Cerebellar Vermis	VIIIa (Vermis) (69); VIIb (Vermis) (29); VIIb (Hem) (1)
8.44	−34	−54	−21	Left Cerebellum	VI (Hem) (20)
8.63	14	−42	−51	Right Cerebellum	VIIIb (Hem) (61); IX (Hem) (28); X (Hem) (5)
5.64	26	−46	−49	Right Cerebellum	VIIIb (Hem) (50); VIIIa (Hem) (28)
**1st order**					
4.81	14	−56	−13	Right Cerebellum	VI (Hem) (83); V (17)
4.2	8	−64	−5	Right Cerebellum	VI (Hem) (2)
4.07	6	−54	−57	Right Cerebellum	IX (Hem) (94); VIIIb (Hem) (2)
**2nd order**					
4.32	2	−64	−41	Cerebellar Vermis	VIIIb (Vermis) (81); IX (Vermis) (16); VIIIa (Vermis) (4)
4.12	−12	−62	−49	Left Cerebellum	IX (Hem) (55); VIIIb (Hem) (18)
**4th order**					
6.39	32	−46	−25	Right Cerebellum	VI (Hem) (44); V (4)
4.61	36	−48	−31	Right Cerebellum	VI (Hem) (88); VIIa Crus I (Hem) (12)
5.74	−4	−60	−1	Left Cerebellum	V (12); VI (Vermis) (3); VI (Hem) (2)
4.93	−18	−64	−25	Left Cerebellum	VI (Hem) (80)
4.75	−6	−70	−7	Left Cerebellum	VI
4.48	−14	−72	−15	Left Cerebellum	VI (Hem) (96)
4.31	−16	−66	−17	Left Cerebellum	VI (Hem) (100)
5.3	−34	−48	−27	Left Cerebellum	VI (Hem) (98); V (2)
5.23	16	−62	−25	Right Cerebellum	VI (Hem) (72); V (4)
4.17	−30	−46	−45	Left Cerebellum	VIIIa (Hem) (4)
4.37	54	−54	−29	Right Cerebellum	VIIa Crus I (Hem)
4.02	0	−72	−33	Cerebellar Vermis	VIIb (Vermis) (51); VIIIa (Vermis) (47); VIIa Crus II (Vermis) (2)
3.98	−24	−58	−49	Left Cerebellum	VIIIa (Hem) (81); VIIIb (Hem) (3)
**−1st order**					
4.06	−48	−42	−39	Left Cerebellum	VIIa Crus I (Hem) (82); VI (Hem) (12)
**−3rd order**					
6.02	18	−56	−23	Right Cerebellum	VI (Hem) (94); V (5)
5.08	26	−54	−25	Right Cerebellum	VI (Hem) (99)
4.45	12	−82	−47	Right Cerebellum	VIIa Crus II (Hem) (59); VIIb (Hem) (41)
4.55	28	−76	−53	Right Cerebellum	VIIb (Hem) (78); VIIa Crus II (Hem) (22)
4.71	10	−64	−11	Right Cerebellum	VI (Hem) (87); V (5)
4.07	34	−46	−29	Right Cerebellum	VI (Hem) (100)

1st order positive linear effects were found in the ipsilateral cerebellum (lobules V‐VI and IX) (Table 2 and Fig. 2). Negative 1st order effects were highly localized to the contralateral cerebellum (Crus I of lobule VIIa and a small part of lobule VI).

**Figure 3:**
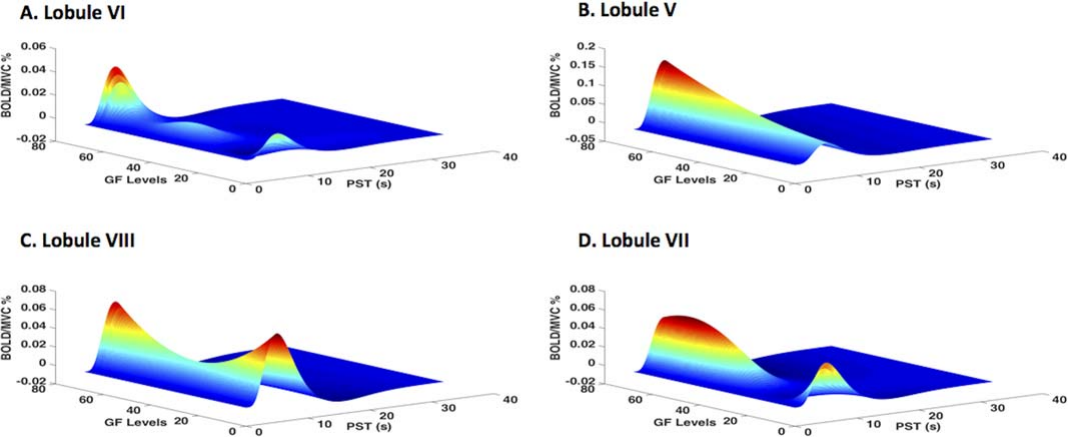
Example of BOLD responses (*Z* axis) based on fitted polynomial functions of GF (*Y* axis) over poststimulus time (PST) (*X* axis). In other words, these graphs show the estimated changes in the haemodynamic (BOLD) response signal per unit change of the regressor coefficient. Importantly, this figure shows the relationships between GF and BOLD response based on all components of the polynomial expansion. This figure shows examples from voxels in different lobules (**A.** VI, **B.** V, **C.** VIII, **D.** VII) based on the group results (i.e., polynomial coefficients and canonical HRF). [Color figure can be viewed at http://wileyonlinelibrary.com]

Higher order positive non‐linear effects were mostly found bilaterally (Table 2 and Fig. 2), especially 4th order effects were found in lobules V‐VI as well as Crus I, contralateral posterior cerebellum (VIIIa and b) and the posterior vermis. The 2nd order effects were more localized in the posterior vermis and lobules VIII and IX. A negative 3rd order effect was detected in the ipsilateral cerebellum (lobules V‐VII and Crus II) (Table 2 and Fig. 2). Exemple responses in different cerebellar areas are provided in Figure 3. This figure illustrates the relationship between the different GF levels and BOLD signals based on all the polynomial orders and each plot represents maximum likelihood estimates of the mapping between BOLD response and GF levels.

**Figure 4:**
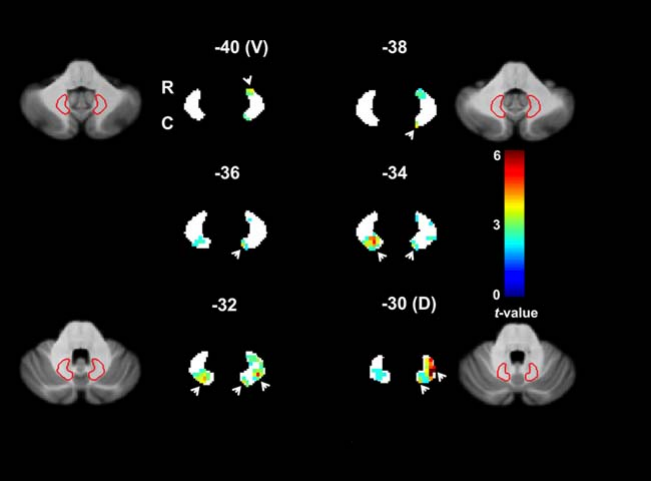
Activations projected onto the dentate nuclei showing examples of the main effect of gripping (i.e., 0th order) using a threshold of *T*‐value ≥ 2, for illustration purposes (significant results are indicated using white arrows in the figure). In these effect maps, right is right (ipsilateral) and all activations are projected on axial sections (white numbers indicate *z*‐coordinate). R: rostral; C: caudal; V: ventral; D: dorsal. [Color figure can be viewed at http://wileyonlinelibrary.com]

**Figure 5:**
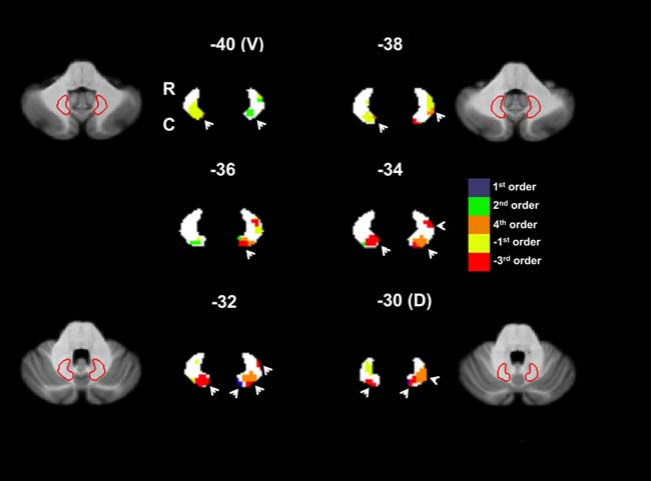
Activations projected onto the dentate nuclei showing examples of force related effects surviving a threshold of *T*‐value ≥ 2, for illustration purposes (significant results are indicated using white arrows in the figure). In the effect maps, right is right (ipsilateral) and all activations are projected on axial sections (white numbers indicate *z*‐coordinate). R: rostral; C: caudal; V: ventral; D: dorsal. [Color figure can be viewed at http://wileyonlinelibrary.com]

Furthermore, we found that the right DN showed a main effect of movement (e.g., 0th order, Fig. 4) as well as higher order non‐linear effects (Fig. 5). For example, during the main effect of movement, all the ipsilateral subdivisions of the DN were involved—with more extensive involvements of the DRDN and DCDN (i.e., more dorsal DN involvements). The contralateral DN was also involved—with activations mainly localized in the DCDN.

When looking at the force related effects, positive effects were mainly localized in the ipsilateral DN; whereas the contralateral DN expressed predominantly negative relationships between GF and BOLD signal. Looking at the subdivisions of the DN, we found that the ipsilateral DCDN showed positive 1st (blue voxels) and 4th order effects—with the 4th order having a wider spatial extent. A 4th order effect was also observed in the ipsilateral DRDN, covering only a small spatial extent. The ipsilateral VCDN mainly showed a positive 2nd effect and a small positive 1st effect. The 2nd order effect was also observed in the ipsilateral DRDN. In addition, activations dominated by negative 1st order behavior were localized in the contralateral VCDN and VRDN. Voxels displaying negative 3rd order effects were localized to the contralateral VCDN and DRDN.

Moreover, results from ART showed that head motion was negligible as translations were one order of magnitude smaller than the voxel size and rotations a fraction of a degree (Supporting Information ‐figures 1). Also no outliers were detected (i.e., no excessive motion or artifacts in the fMRI time series were detected using the ART software). Furthermore, there were no significant correlations between any of the head motion parameters with each GF level. Figures that provide summaries of this analysis are shown in the Supporting Information (figures 1 and 2).

It should be noted that Table 2 includes only the peak significant voxels for each cluster. Figure 2 is complementary to Table 2 as, for example, it can be seen that the top right cluster of the 0th order effect map is highly significant and extends into both the anterior and posterior lobes.

## DISCUSSION

In this work, we have characterized the cerebellar responses during a visually‐guided dynamic grip task (with five different force targets) using a recently proposed pipeline that uses the optimized SUIT template for the cerebellum and DN. The main findings of this study are:

1. A main effect of gripping using the dominant right hand (i.e., the 0th order effect) not only shows, as expected, clear involvement of the anterior lobe but also part of the posterior lobe (bilaterally) and the DN (especially the dorsal sections).
2. An increase in task complexity (i.e., increasing GF levels) has a two‐fold effect: on the one hand, there is increased recruitment (i.e., a positive linear effect) of motor areas of the anterior lobe and, conversely, there is decreased recruitment (i.e., a negative linear effect—indicating reduced BOLD amplitude as the GF increases) in the contralateral posterior lobe (area VII).
3. Non‐linear BOLD responses to GF were observed in bilateral motor, cognitive and associative cerebellar areas as well as in the DN, suggesting that both cerebellar hemispheres may play a role in motor control during visually‐guided gripping, in synergy with the DN. These results also suggest that the DN shows a somatotopic organization that responds non‐linearly, consistent with empirical observations in the cerebellar cortex (from which afferent signals are transmitted) and similarly to the cerebral neo‐cortex (which receives the signals), indicating a consistency between functionally specific motor and associative areas [Alahmadi et al., 2016d].

In order to try to understand the implications of these results for the cerebellum's role in motor control and associative/cognitive functions, we now discuss the grip related effects localized in the anterior (V) and posterior lobes (VI, VII, VIII, IX, X), as well as the DN.

### Anterior Lobe

The anterior lobe of the cerebellum is known to play a key role in sensory‐motor integration and motor control [Stoodley and Schmahmann, 2009]. The main effect of movement results (i.e., the 0th order effect) confirmed these findings; especially in lobule V. A recent meta analysis of the different types of gripping (i.e., power or precision) or different patterns (static or dynamic) showed that lobule V was involved in all these motor tasks [King et al., 2014]; suggesting that this lobule is involved in the basic aspects of motor control during gripping. Within the medial region of lobule V, a positive linear relationship between force levels and activation was detected in our study, in line with previous findings with both low force [Keisker et al., 2009] and higher force ranges [Spraker et al., 2012]. We speculate that this indicates an increased recruitment of neurons in motor regions at the highest forces. In addition to this linear effect, we also observed a non‐linear (−3rd order) effect in part of the anterior lobe (V, laterally). Interestingly, a similar co‐localization of 1st and negative 3rd‐order parametric effects was found in the primary motor cortex (M1) [figure 5a in Alahmadi et al., 2016d]. While this is not an unexpected finding, as the anterior cerebellum and M1 are richly interconnected, the consistent association of 1st/−3rd BOLD/GF relationships in key motor areas suggests that this pattern could represent a signature of motor control activity. The finding that within functionally engaged regions, there are different forms of the BOLD‐GF relationship could reflect different microvascular organizations supporting neuronal activity [Alahmadi et al., 2016d] and/or that different populations of neurons within a certain functional area have different functional roles [Alahmadi et al., 2016d; Ashe, 1997; Ward and Frackowiak, 2003].

### Posterior Lobe

The posterior cerebellum was initially thought to be mostly involved in higher level functions such as working memory, attention and executive functions [Stoodley and Schmahmann, 2009], and in higher‐order motor control, such as the integration of visual cues to guide movement [Balsters et al., 2010; Keisker et al., 2009; Miall et al., 2000; Ramnani, 2006; Ramnani et al., 2001; Vaillancourt et al., 2003]. However, it has been demonstrated that the posterior cerebellum also possesses a secondary representation of the hand, localized in lobule VIII, while a new sensorimotor representation of the hand produced during complex finger tasks has been mapped to lobule VI (laterally) [Grodd et al., 2001; Rijntjes et al., 1999; Schlerf et al., 2010, 2014]. The current study confirms a widespread involvement of the posterior cerebellum, including lobules VII, VIII, IX and the posterior vermis, all ipsilateral to the hand used, and lobule VI bilaterally associated with the main effect of the visually guided dynamic power grip task used in this study. The involvement of lobules VII and IX as well as the contralateral lobule VI can be related to the use of the squeeze ball device, which could represent sensory elements from the fingers and palm. Our paradigm also involves executing a complex dynamic power grip task, requiring cognitive processing, including visuomotor integration [Kuhtz‐Buschbeck et al., 2008], that could modulate cerebellar activity. It is also possible that the widespread activations of the posterior cerebellum are due to the wide range of force levels used in our experimental paradigm; in fact, in a recent power grip study using a very low range of target forces (1–10% MVC), the authors did not detect activations in lobules VII and IX of the cerebellum [Kuhtz‐Buschbeck et al., 2008] while posterior cerebellum activations were noted in other GF studies investigating larger force ranges. In addition, it is common to find overflow of muscle activity from the contracting hand to the contralateral (relaxing) hand. In this context, the decreased linear recruitment (negative 1st‐order effect) found in the posterior lobe of the cerebellum (mainly lobule VII – Crus I) may also indicate a greater effort to achieve suppression of muscle activity of the contralateral hand. This is indeed an interesting observation that needs further investigation.

One of the interesting findings of this study is the bilateral activation in lobule VI seen in the main effect of movement analysis; this is in line with previous GF studies [Halder et al., 2007; Kuhtz‐Buschbeck et al., 2008], although it has rarely been discussed or possibly not detected [Keisker et al., 2009]. Our data, however, not only shows a contralateral activation of lobule VI (as well as the more familiar ipsilateral one) but also shows that the activation pattern of lobule VI is not homogenous. For example, in our study the contralateral lobule VI presents with a purely higher order non‐linear association with GF, while the corresponding ipsilateral area responds with both linear and non‐linear components. It is possible, therefore, that this contralateral response profile is linked to complexity of the task. In addition, considering the potential hypothesis of overflow effects from the ipsilateral to the contralateral side [Addamo et al., 2007; Henry and Smith, 1961], it is also possible that an increase in GF, which requires increased muscle activity, results in an increased BOLD effect in the contralateral side; therefore resulting in increased bilateral activations as shown in lobule VI. Another possible explanation is that this response reflects local inhibition [D'Angelo and Casali, 2013; D'Angelo et al., 2013]; as in some regions where higher‐order effects were detected, a main effect of movement was not found. In a recent study [Kipping et al., 2013], it was shown that lobule VI has functional connections with premotor areas, which we find have a similar response pattern to what we have reported above in lobule VI. This suggests the possible specificity of this parametric profile for higher‐order motor control. Indeed, in accordance with the speculative interpretation that non‐linear associations may represent the functional correlate of higher‐order components of motor control (or areas that are involved in sensory‐motor integration, attention, executive planning) [Alahmadi et al., 2016d; Keisker et al., 2009; Spraker et al., 2012], the meta‐analysis by Stoodley and Schmahmann [2009] found that lobule VI was involved in motor, somatosensory, language, working memory, spatial task, executive and emotional functions. Moreover, the findings reported here suggest that non‐linear responses can be associated not only with visuospatial processing but also with increased attentional demands at very high or low force levels as shown by positive 2nd order and 4th order effects in the inferior posterior lobe and in the superior lobules, respectively. Also, this may reflect metabolically optimal energy consumption at intermediate forces, especially in the 2nd order effect, resulting in a reduced BOLD signal at mid force levels.

Interestingly, these cerebellar areas project to cortical areas (such as the prefrontal cortex, the premotor cortex and the lateral parietal cortices) that show similar GF‐BOLD behavior [Alahmadi et al., 2016d]—and are all involved in attentional modulation of motor activity [Balsters et al., 2010; Keisker et al., 2009; Kelly and Strick, 2003].

One of the interesting findings of this study is the widespread evidence for a negative 3rd order BOLD‐GF effect within the posterior cerebellum (mainly in lobules VI and VII) and part of the anterior cerebellum (lobule V). The biophysical mechanisms behind such a relationship cannot be inferred from the present data, but two plausible explanations are as follows. When considering animal studies showing non‐linear neuronal responses as a function of applied force, it is possible to identify neurometric functions reflecting either saturation, or a reduced firing at high forces or firing patterns that follow an S‐shaped profile [Ashe, 1997; Cheney and Fetz, 1980; Evarts et al., 1983]. The negative 3rd order effect that we have detected could reflect the BOLD response to S shaped neuronal responses. The alternative explanation for our finding is that the negative 3rd order effects are mainly localized within the posterior lobules, which are areas known to be related to visual and associative functions. Thus, the reduced BOLD signal at the highest GF levels may indicate a redistribution of oxygen toward motor‐related areas (characterized by linear or U‐Shaped responses) due to increased metabolic demand at high GFs [Alahmadi et al., 2016d].

Moreover, it is interesting to note that the 1st, 2nd, and −3rd effects define an almost continuous area connecting the anterior and posterior lobules (Fig. 2). This functional anatomy may have various explanations. One possibility is that each of these non‐linear response components in the cerebellar cortex is mediated by a specific connectivity with cerebrocortical areas showing the same non‐linear response, so that the BOLD response simply inherits the activation state of the up‐stream cortical circuit. Another possibility is that local cerebellar processing generates specific spatially organized patterns of excitation and inhibition and thereby of local blood flow, around the lobule VI cluster.

The neurobiological source of the non‐linear behavior still remains to be understood, but its contribution is strongly supported by the data. One potential approach could be to apply additional neurovascular and cerebral quantitative methods or additional higher order fMRI tasks. Distinguishing between these two explanations would require the direct measurement of neurovascular coupling in the cerebellar tissue in response to various patterns of neuronal activity.

### Dentate Nuclei

In this study, we were able to characterize BOLD signal responses in the DN. Responses in these nuclei are not frequently reported, most likely because of their small size and their T2*‐weighted signal variability, which makes their responses difficult to detect [Alahmadi et al., 2015; Diedrichsen et al., 2011; Dimitrova et al., 2006; Habas, 2010; Küper et al., 2011, 2012, 2014]. The data presented here, however, demonstrates that these nuclei show a main effect of movement as well as non‐linear force‐related‐BOLD effects. The DN is connected to the thalamus and, from there, to motor, non‐motor, associative and cognitive cortical regions [Alahmadi et al., 2015; D'Angelo and Casali, 2013; Küper et al., 2011; Palesi et al., 2014], through the superior cerebellar peduncles. In our previous work on force‐mediated‐BOLD cortical brain activations, we observed similar non‐linear behavior in associative parietal regions [Alahmadi et al., 2016d]. To investigate how these non‐linear behaviors are related to DN somatotopic mapping, we relied on published work showing that it is possible to functionally parcellate the DN. Studies of DN efferent connections or functions [Clower et al., 2005; Dum and Strick, 2003; Glickstein et al., 2011; Küper et al., 2011, 2014; Strick et al., 2009] suggest that the dorsal sections of the DN are more related to motor functions; whereas the ventral sections are more related to cognitive and spatial functions (non–motor functions).

For example, the finding in our study that positive non‐linear effects (+2nd) were mainly localized in the ventral section of the DN matches the findings of the same parametric behavior in the posterior parietal lobule [Alahmadi et al., 2016d]. This could imply a functional connection between the ventral DN and the posterior parietal lobule as both demonstrate an involvement in visual related functions [Elsinger et al., 2006; Glickstein et al., 2011; Hamzei et al., 2002].

In addition, using a high field MRI scanner (7T) and in‐vivo sub‐millimetre structural diffusion MRI, Steele et al. [2016] investigated the connections between the cerebellar lobules (IV, V, VI, Crus I, and Crus II) and DN sub‐sections in healthy subjects. After performing lobule‐specific tractography and group classification based on their connection to the DN, they found that lobules IV, V, VI, which were classified in their manuscript as motor lobules, were connected to the superior portion of the DN, while Crus I and Crus II, which were classified by Steele et al. as non‐motor regions, were connected to the posterior and lateral DN as well as to the inferior portion of the DN [Steele et al., 2016]. The study by Steele et al. also found that a larger portion of the DN was connected to the non‐motor lobules, confirming the key role of the cerebellum in cognitive processes. These findings are also in agreement with the work by Strick et al. [Dum and Strick, 2003; Strick et al., 2009]. The white matter structural connectivity work by Steele et al. thus shows that the anterior cerebellum is predominately connected to the superior (dorsal) DN, while the posterior cerebellum is predominately connected to the inferior (ventral) DN [Steele et al., 2016]. It should be noted that Crus I and Crus II, classified as non‐motor lobules in the work of Steele et al., were shown to be involved in motor functions, especially visuomotor tasks, as seen in this study and previous studies [Alahmadi et al., 2016d; Kuhtz‐Buschbeck et al., 2008]. In addition and in line with these findings, a recent resting state fMRI study showed that the superior (dorsal) DN (used as a seed) was principally connected to the anterior cerebellum (I‐V) as well as lobule VI, whereas the inferior (ventral) DN (used as a seed) was connected to the posterior cerebellum (mainly Crus II) [Bernard et al., 2013]. These resting state fMRI findings are highly consistent with previous structural connectivity and animal works [Dum and Strick, 2003; Steele et al., 2016; Strick et al., 2009].

Although our current paradigm is a task specific fMRI study (a visuomotor task), the majority of our findings are in line with the aforementioned data, from a functional segregation point of view. For example, the 0th order (the main effect of gripping) was largely localized (with the largest effect sizes) to the dorsal part of the DN and the anterior lobe of the cerebellum as well as lobule VI. In line with the previous studies, this would be expected because of the motor nature of the task used in this study. The posterior cerebellum and the ventral portion of the DN showed some activations during the main effect of gripping that could be related to the use of the visual cue (a non‐motor domain). The activations in these non‐motor portions of the DN were smaller (in amplitude and extent) as compared to the motor regions in the cerebellum and the DN. Looking at the force related effects and the somatotopic organization of the DN—in relation to cerebellar findings—the organization is mostly in line with previous publications. For example, the −3rd order effect was largely restricted to functionally specific areas of the DN and the cerebellar cortex; that is, mainly within the anterior cerebellar lobe, lobule VI and the dorsal portion of the DN. These areas were shown in previous work to be structurally and functionally connected [Bernard et al., 2013; Dum and Strick, 2003; Steele et al., 2016; Strick et al., 2009]. Importantly, −3rd order effects were also detected in the posterior area of M1 [Alahmadi et al., 2016d], consistent with the known connectivity between the dorsal DN, anterior cerebellum and motor cortical areas [Bernard et al., 2013; Dum and Strick, 2003; Steele et al., 2016; Strick et al., 2009]. Further areas where the −3rd order effect was observed were cerebellar Crus I and Crus II as well as part of the ventral DN. The 4th order effect was detected dorsally and ventrally in the DN and within lobule VI and Crus I in the cerebellar cortex. The 2nd order effect was detected in Crus I and the ventral part of the DN. Most of these findings are in agreement with previous presentations of the cerebral‐cerebellar and DN somatotopic organization [Dum and Strick, 2003; Steele et al., 2016; Strick et al., 2009], suggesting that the DN and cerebellar cortex are functionally related during a visuomotor task. However, there are some differences seen in our study. For example, most of the effects were mainly lateralized to the right hemisphere. Also, the DN has a larger −1st order effect compared to the cerebellum. The +1st order was detected with a larger extent in the anterior cerebellum as compared to the dorsal DN. We believe that these differences are due to the type of the task (visuomotor) (i.e., this study is biased toward visuomotor networks) and to possible loss of sensitivity in the DN due to the intrinsic limitations on the resolution of our fMRI data.

For a complete mapping of the deep cerebellar nuclei interactions, other cerebellar nuclei (such as the fastigial, globose and emboliform nuclei) could be investigated, but despite their important functions and roles, their size is too small to be examined using the images acquired in this study.

In addition, there are methodological considerations that should be taken into account for this and other, similar studies. In our paradigm, an external visual cue was used by subjects to apply accurate forces while gripping. Such methodology has been commonly applied in previous studies [e.g., Alahmadi et al., 2015, 2016d; Galléa et al., 2008; Hilty et al., 2011; Keisker et al., 2009, 2010; Kuhtz‐Buschbeck et al., 2008; Neely et al., 2013; Spraker et al., 2007, 2009, 2012; Sulzer et al., 2011; Vaillancourt et al., 2003; Ward, 2004; Ward and Frackowiak, 2003; Ward et al., 2008; Wong et al., 2007]. These studies referred to such networks as visually guided motor networks or visuomotor networks.

It is believed that such paradigms mimic the functions of everyday life. Such networks are used daily, and are therefore worthy of investigation in their own right. Distinguishing visual and motor effects is not possible with these paradigms. To separate these contributions, an additional paradigm would probably be needed. For example, the visual cues could be presented without the need of gripping or the same paradigm could be used without the visual feedback. This, however, would have the drawback of making the interpretation of the GF‐BOLD relationships harder to achieve.

Other studies have tried to separate visual from motor functions [e.g., Kuhtz‐Buschbeck et al., 2008; Man et al., 2015; Noble et al., 2013; Vaillancourt et al., 2003]. Although the cerebellum was not the focus of these studies (and thus their pipeline was not optimized to detect effects in this region), Kuhtz‐Buschbeck et al. (for example) showed activations in the posterior cerebellum (lobule VI) with visuomotor stimuli, but could not detect activations in this area with motor tasks only. Also, the authors found that in the presence of visual cues, the precision task activated only Crus I, while the power task activated only lobule VI. Moreover, Vaillancourt et al. [2003] investigated the effects of the absence or presence of a visual cue during a static precision task. With respect to cerebellar activations, the authors showed that the posterior cerebellum and the dentate nucleus were directly involved during the visuomotor task. Based on these findings, we could hypothesize that the posterior cerebellum, activated in our study, was more related to the visuomotor integration process. Future studies, purposely designed, may be able to investigate and distinguish visuomotor, visual and motor processing with respect to the relationship of the GF‐BOLD signal.

When comparing our results with previous studies, it should be noted that only a few studies have characterized non‐linear responses in vivo in humans and these non‐linearities were rarely fitted to specific functions or reported within the cerebellar lobules. For example, Keisker et al. [2009] showed (without any parametric fitting) that the posterior cerebellum exhibited a greater response at low and high forces; whereas it responded with a lower BOLD signal at middle forces, indicating a match to the +2nd order fit of our study. However, Keisker's study used only three forces, precluding the characterization of higher‐order non‐linearities. In addition, Spraker et al. [2012] showed that the relationship between BOLD and applied force tended to covary non‐linearly in most of the posterior lobules. They did not fit the data using non‐linear functions, but reported that the percentage signal change in these regions indicated a signal reduction corresponding to the highest force levels. This behavior could potentially be captured by a negative 1st or 3rd order polynomial function. Ultimately, the strongest argument for the use of polynomial functions is that in animal studies, recordings of neuronal cell firing during different movement tasks show relationships that assumed linear, exponential and sigmoid (S) shapes [Ashe, 1997; Cheney and Fetz, 1980; Conrad et al., 1977; Evarts et al., 1983; Georgopoulos et al., 1992; Hepp‐Reymond et al., 1994; Maier et al., 1993; Taira et al., 1996].

One interesting issue—that could be further investigated—would be to assess differences in post stimuli time (PST) and reaction times, as well as characterizing haemodynamic delays using a temporal basis set for the HRF [Friston et al., 2000]. However, the paradigm used in this study was optimized to detect changes in the amplitude of the BOLD signal, making the characterization of the HRF and PST challenging tasks with the current setting—due to the short trial durations.

It should be noted that areas showing a significant BOLD‐GF relationship do not necessarily have to show a 0th order effect. When this occurs, it means that the BOLD signal across all trials (i.e., all GFs) is not significantly different from baseline, despite showing a specific modulation with respect to the applied different GFs. For example, if an area has a significant positive linear response but does not have a significant 0th order effect, it produces a negative BOLD response when applying low forces that becomes positive when applying high forces. Such a behavior may suggest an interplay between excitatory and inhibitory neurons, which is force‐dependent. Interestingly, inhibition and negative BOLD signal have been observed previously, although not in relation to variable applied GFs [Logothetis, 2008; Logothetis et al., 2001; Newton et al., 2005; Singh, 2012].

In addition, head motion was shown to be negligible in this cohort and not correlated to any of the GF levels. Nevertheless, we adjusted the GLM regression model by entering motion parameters as regressors of no interest [Friston et al., 1996; Johnstone et al., 2006]. Our additional analysis performed using the ART software did not reveal extensive head motion in any direction nor detected any outlier linked to head motion. Thus, we conclude that head motion‐related artifacts on our data are likely to be negligible and that in this study activations were not significantly influenced by head motion.

## CONCLUSION

The study demonstrates that, using a dedicated analysis protocol, linear and non‐linear BOLD responses to motor task performance are found in cerebellar grey matter structures—and that the parametric form of these responses can be characterized using a dedicated protocol. We have demonstrated linear and non‐linear BOLD responses to GF modulation with a clear and distributed regional specificity in the cerebellar lobules and nuclei. Crucially, these reflect our previous findings in the cerebral cortex, with 0th order and linear effects in motor regions and higher order parametric responses in associative and cognitive regions. These findings contribute substantially to our understanding of the cerebellum, beyond a simple motor controller and could have clinical implications if found to be systematically altered in neurological diseases. Our results indicate that cerbellar processing—even in motor tasks—goes beyond its traditional roles.

## Supporting information

Supporting Information
