# Supplementary material for: Cerebellar lobules and dentate nuclei mirror cortical force‐related‐BOLD responses: Beyond all (linear) expectations
Source: Hum Brain Mapp. 2017 Feb 27;38(5):2566–79. doi: 10.1002/hbm.23541 (PMC5413835; doi:10.1002/hbm.23541)

Supplementary material – Figure 1: Group averaged head motion parameters of translations (a) and rotations (b). In (c) the fMRI paradigm is shown displaying also the different force levels.


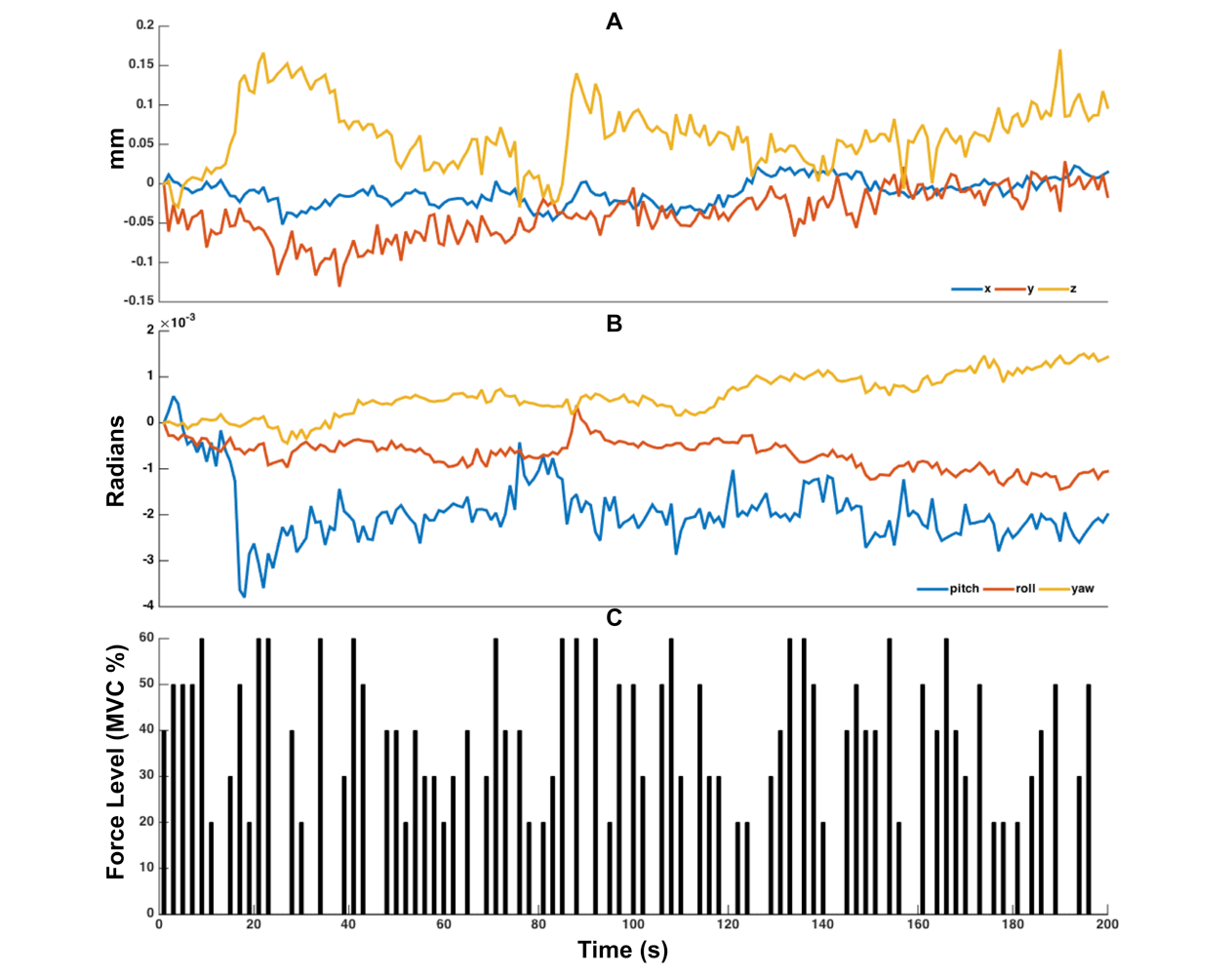


Supplementary material – Figure 2: Correlation matrices showing within subject correlation coefficient (r-values) of head motion parameters (column) and different force level (rows).


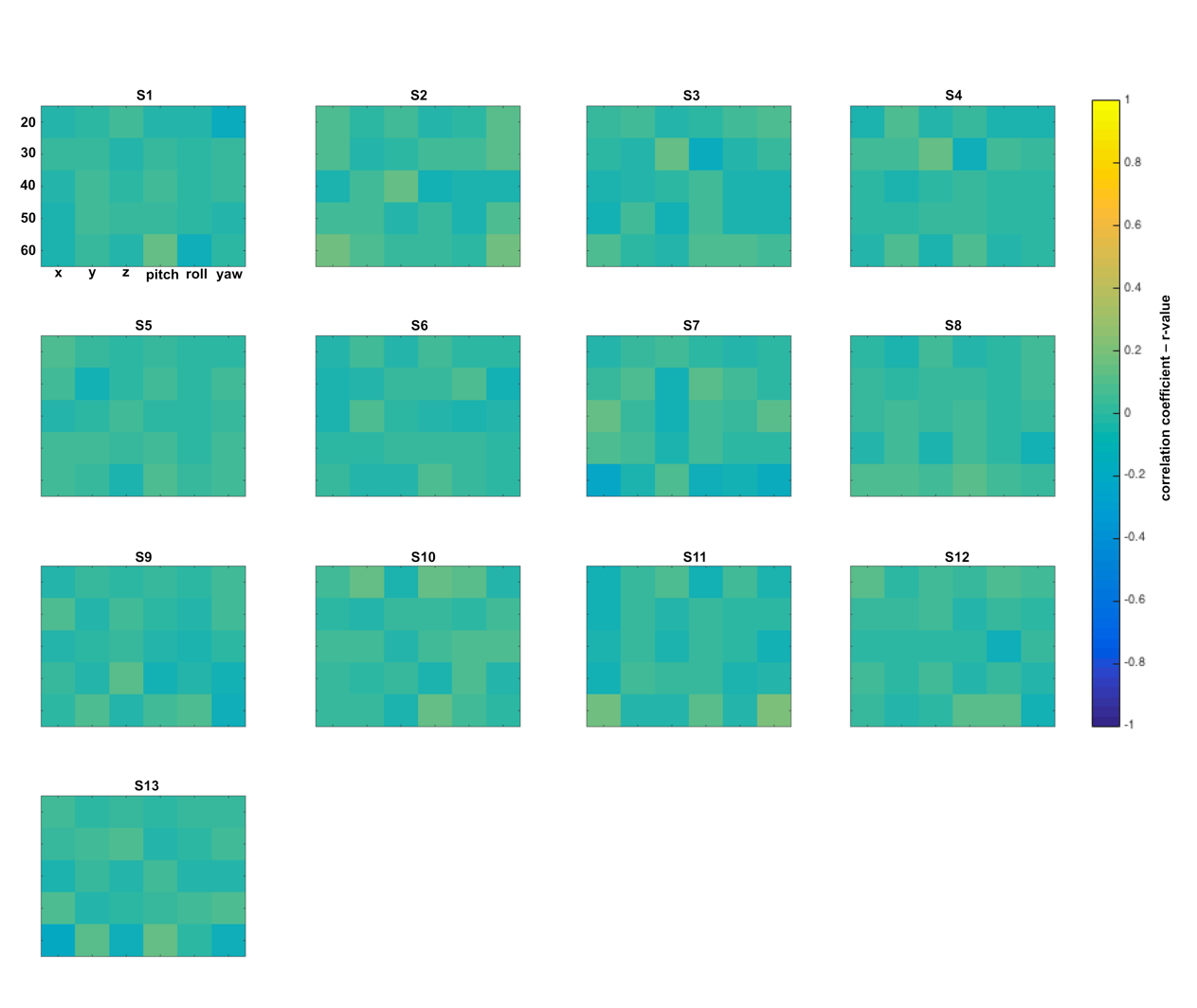

Supplement: Supplementary file 1 — Supporting Information [file HBM-38-2566-s001.docx]
